# STAT1/IFIT2 signaling pathway is involved in PD-L1-mediated epithelial-to-mesenchymal transition in human esophageal cancer

**DOI:** 10.1007/s12094-021-02743-1

**Published:** 2022-02-02

**Authors:** J. Chen, Y. Liu, Y. Zhu, Y. Chen, J. Feng, T. Jiang, X. Zheng, L. Chen, J. Jiang

**Affiliations:** 1 Department of Tumor Biological Treatment, The Third Affiliated Hospital of Soochow University, Jiangsu, Changzhou, 213003 China; 2 Jiangsu Engineering Research Center for Tumor Immunotherapy, Jiangsu, Changzhou, 213003 China; 3 Institute of Cell Therapy, Soochow University, Jiangsu, Changzhou, 213003 China; 4 Department of Neurosurgery, Soochow University, Jiangsu, Changzhou, 213003 China

**Keywords:** PD-L1, IFIT2, EMT, Esophageal cancer

## Abstract

**Background:**

We have previously reported significant change of epithelial to mesenchymal transition (EMT) phenotype of Eca-109 cells upon PD-L1 operation, and the cytoplasmic domain of PD-L1 played an essential role in promoting EMT of esophageal cancer cells. However, the underlying mechanism of how PD-L1 regulated EMT in esophageal cancer remained unclear.

**Methods:**

The overexpression and knockdown expression models of PD-L1 and IFIT2 were established by using lenti-virus transfection and RNAi method. Western blotting, qRT-PCR, CCK8 assay, transwell assay and wound healing assay were chosen to investigate their impact on the cells. The expression levels of IFIT2 and EMT markers in esophageal cancer tissues were examined by immunohistochemical staining. The rescue experiments were further applied to investigate the role of STAT1/IFIT2 signal pathway in the PD-L1-mediated EMT. Luciferase reporter assays were performed to examine the IFIT2 promoter activities upon knockdown expression of PD-L1 to identify the putative targeted region of IFIT2 promoter.

**Results:**

The STAT1/IFIT2 signal pathway was activated when PD-L1 was knockdown in human esophageal cancer cells. Decreased IFIT2 expression significantly increased the cellular abilities of viability, invasion and migration by using RNAi method in human esophageal cancer cells. Decreased IFIT2 expression in esophageal cancer tissues significantly correlated with EMT status, and could be used as an independent prognostic predictor for the patients. Rescue experiments in PD-L1 knockdown cells further confirmed that STAT1/IFIT2 pathway was involved in the PD-L1 mediated EMT of esophageal cancer cells. Moreover, the luciferase reporter assay also confirmed that in esophageal cancer cells, the promoter region of IFIT2 (-3K~-1K) remains more active in PD-L1 knockdown expression cells compared with controls.

**Conclusion:**

Our present work reveals a novel mechanism of how PD-L1 regulates EMT of cancer cells, namely STAT1/IFIT2 signal pathway is required in PD-L1 mediated EMT in human esophageal cancer.

## Background

PD-L1, also known as B7-H1, has been widely accepted as an essential tumor marker as well as an immune therapeutic target for human cancers [1, 2]. Numerous studies have also shown that, besides its negative effect on T cell immune response, abnormal PD-L1 expression also plays an essential role in malignant transformation, such as epithelial-to-mesenchymal transition (EMT) of tumor cells in many human cancers [3]. Azuma et al. have first established the theory that PD-L1 can serve as a bidirectional regulator, the extra-cellular domain of PD-L1 can interact with its receptor PD-1 on T cells, resulting in dampening T-cell mediated anti-tumor response, and the cytoplasmic domain of PD-L1 can also trigger the cellular signaling pathways involved in the anti-apoptosis via ligation with PD-1 fusion protein [4]. Moreover, it has been demonstrated that, PD-L1 transgenic-derived keratinocytes and squamous cell carcinoma cells exhibit a marked reduction of E-cadherin, and elevated expression of the transcription factors Slug and Twist, suggesting that the over-expression of PD-L1 in keratinocytes can promote EMT and accelerate carcinogenesis [5].

We have previously reported that PD-L1 can be used as an important prognostic predictor in human esophageal cancer, and confirmed that PD-L1 can potentially contribute to the EMT of esophageal cancer cells by successfully establishing cellular models including PD-L1 knockdown expression and PD-L1 over-expression in Eca-109 cell line [6]. Moreover, we have found significant change of EMT phenotype of Eca-109 cells upon operation of PD-L1, and the cytoplasmic domain of PD-L1 molecule plays a decisive role in promoting EMT of esophageal cancer cells [6]. However, the detailed molecular mechanism of how PD-L1 regulates EMT of cancer cells still remains largely unexplored. Fei et al. have reported that, over-expression of PD-L1 in nasopharyngeal cancer cells can prominently activate the EMT via PI3K/AKT signaling pathway, conferring malignancy and aggressiveness of nasopharyngeal cancer [7]. Xu et al. have demonstrated that, PD-L1 can induce EMT and enhance stemness through up-regulation of SREBP-1c in human renal cell carcinoma cells [8].

In our present study, we found that modification of PD-L1 expression could significantly regulate the STAT1/IFIT2 signaling pathway in human esophageal cancer cells. IFIT2 is an important member of IFIT family genes which are well known as a group of interferon-stimulated genes (ISGs), thus IFIT2 was also named as ISG54 [9]. IFIT2 has been confirmed to play an important role in suppressing proliferation and migration of cancer cells, and regulation of viral replication, showing anticancer effects and IFN-mediated antiviral effects [10, 11]. Herein, we further investigated the clinical significance and biological role of IFIT2 in human esophageal cancer, and confirmed that STAT1/IFIT2 pathway was involved in the PD-L1-mediated EMT of esophageal cancer cells.

## Materials and methods

### Patients and tissue microarray preparation

Formalin-fixed, paraffin-embedded esophageal cancer tissue samples were collected from 105 patients who underwent surgical resection between February 2005 and May 2006 in our hospital (82 males and 23 females; median age at diagnosis was 59 years). Moreover, five normal tissues from the non-malignant portion of esophagus were collected and used as controls. No patients received pre-operative chemotherapy or radiotherapy. All tumor tissues were confirmed as the esophageal squamous cell carcinoma using hematoxylin and eosin (H&E) staining after surgical resection. All these tissues were used in the construction of tissue microarray. In brief, the H&E-stained standard slides were reviewed from each section of esophageal cancer tissues, and a representative tumor region and the corresponding formalin-fixed paraffin-embedded tissue block were selected for the tissue microarray. The viable invasive carcinoma tissue (epithelial cells) and surrounding tumor stroma from central parts within the tumors were carefully selected and marked on the H&E slides, and then were sampled for the tissue microarray block which was assembled using a tissue-arraying instrument (Beecher Instruments, Silver Springs, MD, USA). Incomplete tissue samples and several missing tissue points were excluded during the heat-induced antigen retrieval, and finally a total of 99 cases of cancer tissues were included in the present statistical analysis. The detailed clinical parameters of the patients are shown in Table 2. The tumor-node-metastasis (TNM) stages were assigned according to the American Joint Committee on Cancer criteria. Among all these 99 patients, the survival data of 86 patients were available. The present study was approved by the ethics committee of our hospital.

### Antibodies and major regents

Rabbit anti-human PD-L1 monoclonal antibody (ab205921), rabbit anti-human IFIT2 polyclonal antibody (ab113112), rabbit anti-human GAPDH monoclonal antibody (ab205921), mouse anti-human STAT1 monoclonal antibody (ab3987) and mouse anti-human STAT1 (phospho Y701) monoclonal antibody (ab29045) were purchased from Abcam (Cambridge, MA, USA). Mouse anti-human E-cadherin (Cell Signaling, Danvers, MA, USA), rabbit anti-human N-cadherin and rabbit anti-human Zeb1 (Santa Cruz, Dallas, TX, USA), goat anti-mouse IgG and goat anti-rabbit IgG (Sigma, St. Louis, MO, USA) were used for Western blotting analysis. Mouse anti-human E-cadherin (MAB-0589) and Vimentin (MAB-0178) monoclonal antibodies used for the immunohistochemistry assay were purchased from Maixin Biotechnology (Fuzhou, China). The HRP-labeled goat anti mouse/rabbit secondary antibodies (K500711) were purchased from Dako (Glostrup, Denmark). The RNeasy Mini Kit was purchased from Qiagen (Valencia, CA, USA), and SYBR Green Master Mix kits were purchased from TaKaRa (Dalian, China). AG490 (S1143, Selleck) were purchased from Selleck (Shanghai, China). RPMI-1640 medium and fetal bovine serum (FBS) were purchased from Gibco (Cambrex, MD, USA).

### Cell lines and cell culture

Human esophageal cancer cell lines, Eca-109 and TE-1 were purchased from Chinese Academy of Sciences, Shanghai Institutes for Biological Sciences (Shanghai, China), and were cultured in RPMI1640 medium supplemented with 10% FBS in the presence of benzylpenicillin (100 U/mL), streptomycin (100 μg/mL) and 2 mM l-glutamine. The cells were incubated under standard culture conditions (5% CO_2_, 37 °C). Moreover, cellular models of the knockdown expression of PD-L1, the over-expression of PD-L1, the knockdown expression of IFIT2, and the corresponding control cells, were established using lenti-virus transfection as previously reported [6, 12–14].

### Immunohistochemistry and the assessment of immunostaining intensity

Immunohistochemical staining was performed as described in our previous studies [6, 12, 15]. Briefly, the consecutive sections from the esophageal cancer tissue array block were prepared, and were dewaxed in xylene, rehydrated and graded ethanol solutions. Antigen retrieval was performed by heating the tissue sections at 100 °C for 30 min in EDTA solution (pH 9.0) or in citrate solution (pH6.0) when needed. The sections were incubated with primary antibodies against PD-L1, IFIT2, E-cadherin and Vimentin, respectively, at 4 °C overnight, followed by incubation with HRP-conjugated secondary antibody. Diaminobenzene was used as the chromogen, and hematoxylin was used as the nuclear counterstained. Finally the sections were dehydrated, cleared and mounted. Moreover, the immunostaining intensity of PD-L1, IFIT2, E-cadherin and Vimentin was evaluated as previously described [6, 12, 15, 16].

### Real-time polymerase chain reaction (RT-PCR)

IFIT2 expression at mRNA level was examined using real-time PCR. In brief, total RNA from the cell lines was extracted using TRIzol (Invitrogen), and PCR reactions were performed using the ABI 7600 system (Applied Biosystems, USA) according to the manufacturer’s instructions. Primer sequences for the detection of the reference gene (GAPDH) and the target gene (IFIT2) were synthesized and listed as follows: human GAPDH forward primer: 5ʹ-TGACTTCAACAGCGACACCCA-3ʹ, human GAPDH reverse primer: 5ʹ-CACCCTGTTGCTGTAGCCAAA-3ʹ, human IFIT2 forward primer: 5ʹ-GCGAAACAACTGCTCCATCT-3ʹ, human IFIT2 reverse primer: 5ʹ-CCAAGACATGCAAAGCCTCA-3ʹ. The relative expression of the target gene was calculated with the 2^–ΔΔCT^ method.

### Western blotting analysis

The Western blotting analysis was used to examine the protein expression levels of PD-L1, STAT1, pSTAT1-y701, IFIT2 and GAPDH. In brief, whole cell extracts were prepared from 1 × 10^6^ cells using RIPA lysis buffer (50 mMTris/HCl pH 7.4, 150 mMNaCl, 1% Nonidet P-40, 0.25% Na-eoxycholate, 1 mM EDTA and protease inhibitor cocktail). Cells were lysed on ice for 30 min, and the lysates were collected in micro-tubes and centrifuged at 12,000 rpm for 15 min at 4 °C. After centrifugation, supernatants were collected and the protein concentrations were measured using a BCA Protein Assay Kit (Beyotime, Jiangsu, China). Equal amounts of denatured proteins were subjected to SDS-PAGE and transferred onto PVDF membranes (Millipore). The membranes were blocked with 5% non-fat dry milk in TBS-T (20 mM Tris, pH 7.4, 137 mM NaCl, 0.05% Tween-20) at room temperature for 3 h, followed by incubation with primary antibodies at 4 °C overnight. Subsequently, blots were washed and incubated with anti-rabbit or anti-mouse secondary antibodies for 1 h. Finally, immuno-reactive protein bands were visualized using an Odyssey Scanning system (Li-Cor, Lincoln, NE, USA).

### Cellular studies of proliferation, invasion and migration

The cell proliferation ability was examined using Cell Counting Kit-8 (CCK-8), the cell migration ability was assessed using a wound healing assay, and the cell invasive ability was examined using matrigel-coated invasion chambers as reported in our previous studies [6, 12, 13, 15].

### Luciferase reporter assay

Luciferase reporter assays were performed to examine the IFIT2 promoter activities upon knockdown expression of PD-L1 to identify the putative targeted region of IFIT2 promoter. Six fragments of the IFIT2 promoter region were amplified and cloned into the pGL3-Basic vector (Promega, Madison, WI, USA). These constructed plasmids were named as pGL3-0.5 k-luc (500 base pairs [bp]), pGL3-1.0 k-luc (1000 bp), pGL3-1.5 k-luc (1500 bp), pGL3-2.0 k-luc (2000 bp), pGL3-2.5 k-luc (2500 bp), and pGL3-3.0 k-luc (3000 bp), according to their sequence lengths. The constructed plasmids were co-transfected with the pRL-TK plasmid into cells using Lipofectamine 3000 (Invitrogen, Carlsbad, CA, USA). The pRL-TK plasmid harboring Renilla luciferase was used to correct the differences in both transfection and harvest efficiencies. The activity of the firefly luciferase reporter carrying IFIT2 promoter was normalized to the Renilla luciferase activity.

### Statistical analysis

GraphPad Prism 5.0 software package (GraphPad Software, Inc., San Diego, USA) was used in the present statistical analysis, and the Student’s *t* test, the Chi-square test or the Log-rank survival analysis was used where appropriate. A *P* value < 0.05 was considered as statistically significant.

## Results

### Response of STAT1/IFIT2 pathway upon PD-L1 operation in human esophageal cancer cell line Eca-109

**Fig. 1:**
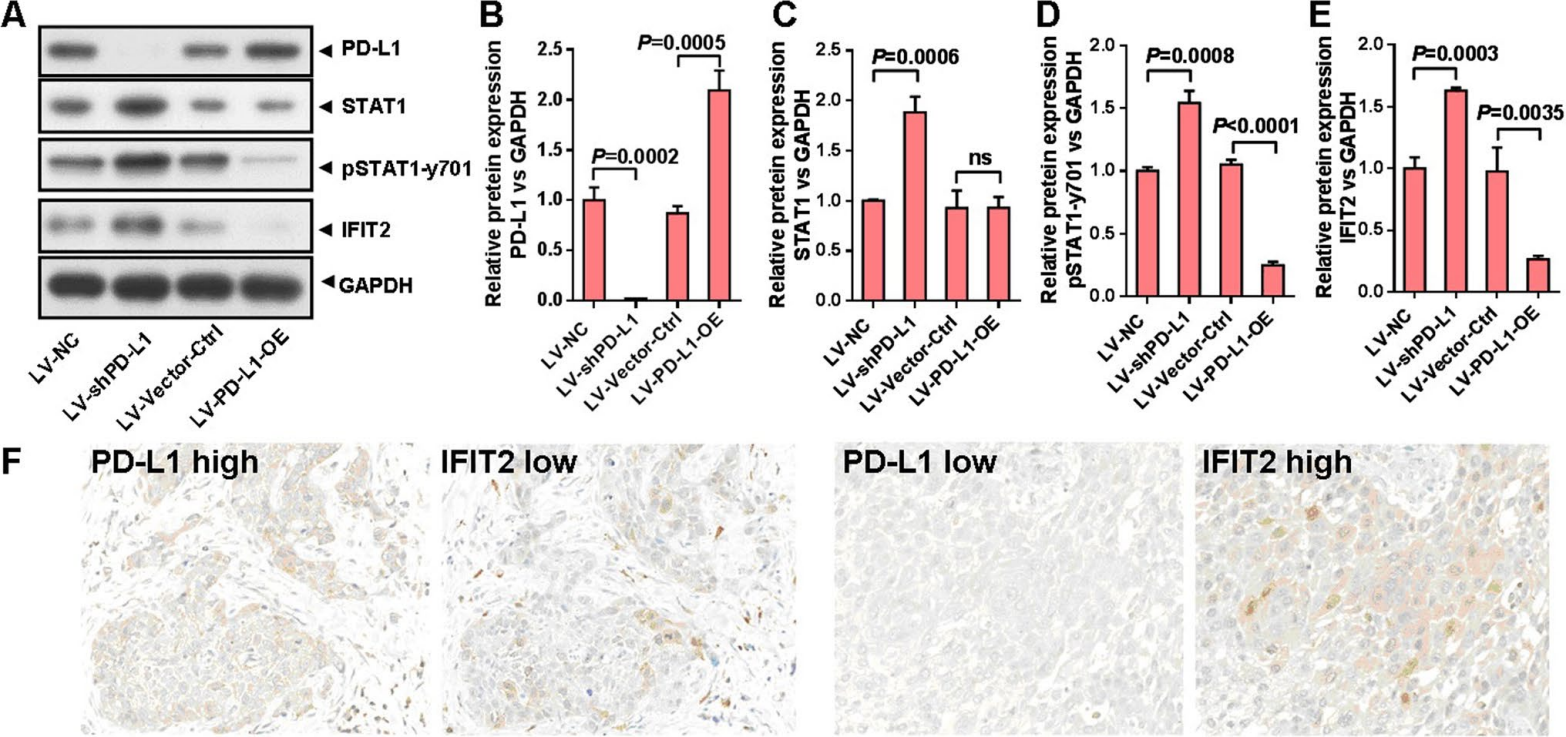
STAT1 and IFIT2 expressions upon PD-L1 operation in human esophageal cancer cell line Eca-109. **A** In different cellular models including LV-NC (negative control for PD-L1 knockdown expression), LV-shPD-L1 (PD-L1 knockdown expression using RNAi method), LV-Vector-Ctrl (negative control for PD-L1 over-expression), and LV-PD-L1-OE (PD-L1 over-expression by transfecting full-length of PD-L1), the Western blotting was used to analyze PD-L1, total STAT1, pSTAT1-y701 and IFIT2 expression. **B** PD-L1 expression level was examined in different cellular models. **C** Total STAT1 was significantly up-regulated in LV-shPD-L1 cells compared with LV-NC cells (*P* = 0.0006), but no difference was found between LV-Vector-Ctrl and LV-PD-L1-OE cells. **D** pSTAT1-y701 was significantly up-regulated in LV-shPD-L1 cells compared with LV-NC cells (*P* = 0.0008), and was significantly down-regulated in LV-PD-L1-OE cells compared with LV-Vector-Ctrl cells (*P* < 0.0001). **E** IFIT2 was significantly up-regulated in LV-shPD-L1 cells compared with LV-NC cells (*P* = 0.0003), and was significantly down-regulated in LV-PD-L1-OE cells compared with LV-Vector-Ctrl cells (*P* = 0.0035). **F** Immunohistochemistry results showed that the expressions of PD-L1 and IFIT2 were negatively correlated, which was found in the consecutive sections of esophageal cancer tissues

In our previous study, we successfully established the cellular models including PD-L1 knockdown expression and PD-L1 over-expression, and further confirmed that PD-L1 positively promotes EMT of esophageal cancer cells [6]. Then, in our present study, we aimed to compare the response of STAT1/IFIT2 signaling pathway among different cellular models. As shown in Fig. 1A, in Eca-109 cells, we performed Western blotting to analyze the expressions of PD-L1, total STAT1, pSTAT1-y701 and IFIT2 in different models including LV-NC (negative control for PD-L1 knockdown expression), LV-shPD-L1 (PD-L1 knockdown expression using RNAi method), LV-Vector-Ctrl (negative control for PD-L1 over-expression), and LV-PD-L1-OE (PD-L1 over-expression by transfecting full-length of PD-L1), respectively. Figure 1B confirms that PD-L1 is down-regulated or up-regulated upon knockdown expression or over-expression of PD-L1, respectively. Figure 1C shows that total STAT1 is significantly up-regulated in LV-shPD-L1 cells in contrast to LV-NC cells (*P* = 0.0006), but no difference between LV-Vector-Ctrl and LV-PD-L1-OE cells. Figure 1D shows that pSTAT1-y701 is significantly up-regulated in LV-shPD-L1 cells in contrast to LV-NC cells (*P* = 0.0008), and was significantly down-regulated in LV-PD-L1-OE cells in contrast to LV-Vector-Ctrl cells (*P* < 0.0001). Figure 1E shows that IFIT2 was significantly up-regulated in LV-shPD-L1 cells in contrast to LV-NC cells (*P* = 0.0003), and was significantly down-regulated in LV-PD-L1-OE cells in contrast to LV-Vector-Ctrl cells (*P* = 0.0035). As shown in Fig. 1F and Table 1, we also revealed that, in human esophageal cancer tissues, the PD-L1 expression level was significantly and inversely associated with IFIT2 expression level (*χ*^2^ = 4.980, *P* = 0.0256).

**Table 1:** Correlation between IFIT2 expression and PD-L1 expression levels in human esophageal cancer tissues

	PD-L1 low (H-score ≤ 140)	PD-L1 high (H-score˃140)	*χ*^2^	*P* value
IFIT2 low (H-score ≤ 25)	8	9	4.980	**0.0256**
IFIT2 high (H-score > 25)	61	21		

Bold signifies *P* < 0.05

### Expression pattern of IFIT2 in human esophageal cancer tissues and the clinical significance

**Fig. 2:**
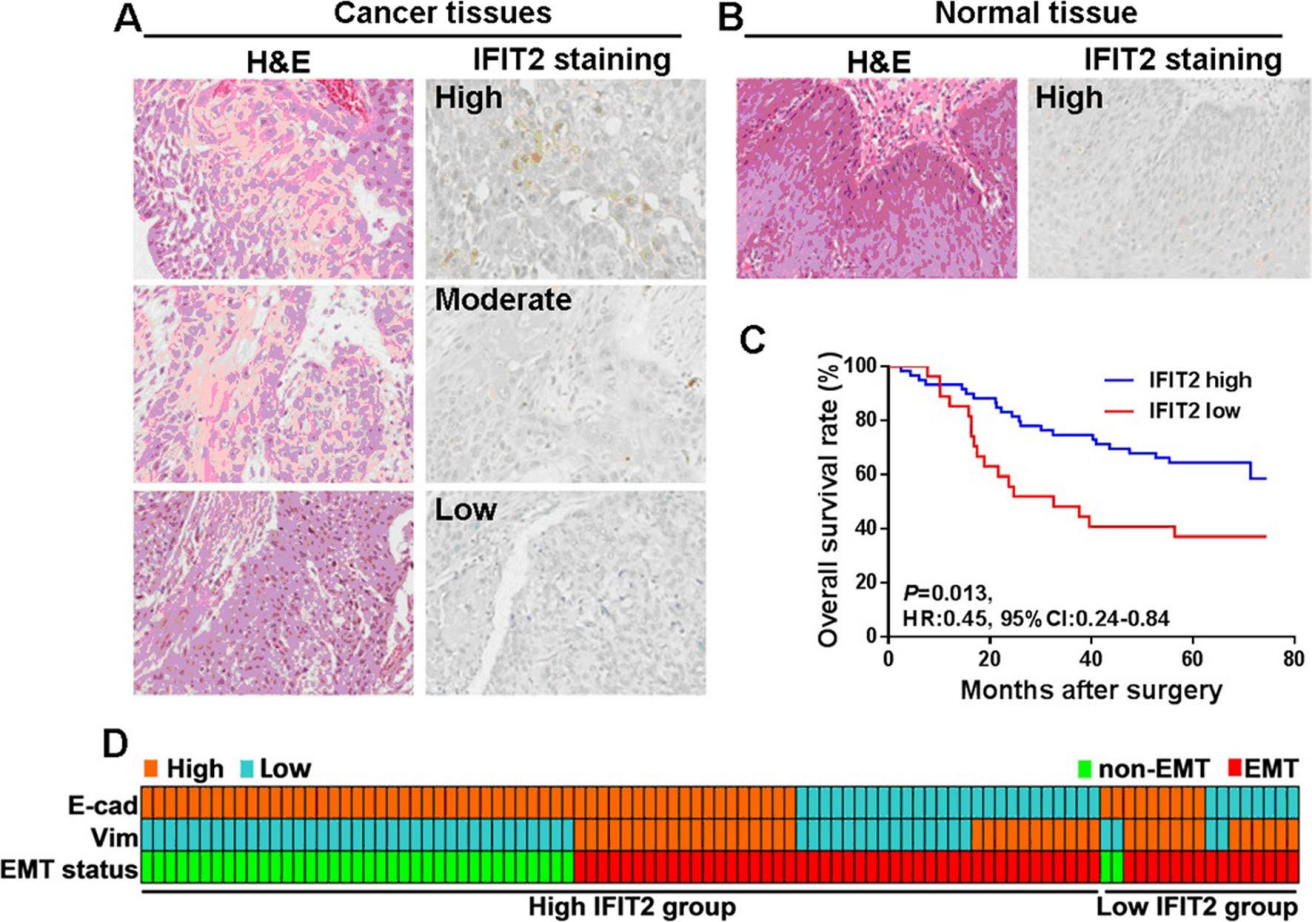
IFIT2 expression in human esophageal cancer tissues. **A** IFIT2 immunostaining could be found in the cytoplasm of cancer cells. **B** High IFIT2 expression could be found in adjacent normal tissue. **C** Survival analysis showed that, the overall survival rate of the patients with high IFIT2 expression was better than that in patients with low IFIT2 expression (HR 0.45, 95% CI 0.24–0.84, *P* = 0.013). **D** We further used the E-cadherin in combination of Vimentin expression levels to characterize all 99 patients into EMT subgroup and non-EMT subgroup. The IFIT2 high-expression group contains 37 cases of non-EMT and 45 cases of EMT, and the IFIT2 low-expression group contains two cases of non-EMT and 15 cases of EMT

The data from our and other groups have confirmed that IFIT2 can serve as an important tumor suppressor gene, and decreased expression of IFIT2 significantly associated with cancer progression and poor prognosis of the patients [10, 12, 15]. Herein, we also aimed to examine the expression pattern and the clinical significance of IFIT2 in human esophageal cancer tissues. As shown in Fig. 2A, IFIT2 immunostaining could be found in the cytoplasm of cancer cells. Moreover, high IFIT2 expression could be found in normal tissues (Fig. 2B). As shown in Fig. 2C, the survival analysis showed that, the overall survival rate of the patients with high IFIT2 expression was better compared with the individuals with low IFIT2 expression (HR 0.45, 95% CI 0.24–0.84, *P* = 0.013). As shown in Table 2, the IFIT2 expression level in esophageal cancer tissues significantly correlates with patient’s gender (*P* = 0.002), but not other parameters, such as age, tumor size, T stage, lymphatic metastasis, distant metastasis or TNM stage. As shown in Table 3, the uni-variate analysis suggested that T stage (*P* = 0.031), lymphatic metastasis (*P* = 0.023), distant metastasis (*P* = 0.039), TNM stage (*P* = 0.050), IFIT2 expression (*P* = 0.013) could be used to predict the patient’s survival, respectively. However, in the multivariate analysis, only IFIT2 expression could be used to independently predict the patient’s survival (HR 0.41, 95% CI 0.19–0.88, *P* = 0.023). As shown in Fig. 2D, if we categorized all the 99 cases of esophageal cancer patients into EMT subgroup (*n* = 60) and non-EMT subgroup (*n* = 39) based on the evaluation of E-cadherin as well as Vimentin expression levels. We found that in IFIT2 higher expression group, there are 37 cases of non-EMT and 45 cases of EMT, and in IFIT2 lower expression group, there are two cases of non-EMT and 15 cases of EMT, which indicates that decreased IFIT2 expression in human esophageal cancer tissues significantly associated with EMT status (*χ*^2^ = 6.562, *P* = 0.0104).

**Table 2:** Correlation between the IFIT2 expression in human esophageal cancer tissues and patients’ clinical parameters

Clinical parameters	Cases	IFIT2 expression level		*χ*^2^	*P* value
		H-score < 50	H-score ≥ 50		
Gender					
Male	76	20	56	9.349	**0.002**
Female	23	14	9		
Age (years)					
≥ 60	51	20	31	1.107	0.293
< 60	48	14	34		
Tumor size					
< 3.5	35	14	21	0.768	0.381
≥ 3.5	64	20	44		
T stage (T)					
T1	8	2	6	0.309	0.578
T2	27	10	17		
T3	45	14	31		
T4	19	8	11		
Lymphatic metastasis ( N)					
Yes	34	15	19	2.194	0.139
No	65	19	46		
Distant metastasis (M)					
Yes	6	2	4	0.003	0.957
No	93	32	61		
TNM stage					
I	5	0	5	1.523	0.217
II	57	19	38		
III	31	13	18		
IV	6	2	4		

Bold signifies *P* < 0.05

**Table 3:** Cox model analysis for the association between the IFIT2 expression level and patient’s clinical parameters in esophageal cancer

Variables	Uni-variate analysis		Multivariate analysis	
	HR (95% CI)	*P*	HR (95% CI)	*P*
Gender (Male: Female)	0.77 (0.38–1.55)	0.465	1.24 (0.53–2.91)	0.620
Age (≥ 60: < 60 years)	0.90 (0.48–1.69)	0.742	1.14 (0.57–2.25)	0.714
Tumor size (≥ 3.5 cm: < 3.5 cm)	1.24 (0.63–2.44)	0.543	1.00 (0.48–2.08)	0.992
T stage (T3–T4:T1–T2)	2.28 (1.08–4.80)	**0.031**	2.18 (0.87–5.47)	0.096
Lymphatic metastasis (Yes: No)	2.08 (1.11–3.90)	**0.023**	1.89 (0.66–5.44)	0.239
Distant metastasis (Yes: No)	3.00 (1.06–8.48)	**0.039**	2.19 (0.67–7.18)	0.194
TNM stage (III + IV: I + II)	1.88 (1.00–3.53)	**0.050**	0.65 (0.19–2.18)	0.484
IFIT2 expression (High: Low)	0.45 (0.24–0.84)	**0.013**	0.41 (0.19–0.88)	**0.023**

Bold signifies *P* < 0.05

### Knockdown of IFIT2 expression in human esophageal cancer cell lines Eca-109 and TE-1

**Fig. 3:**
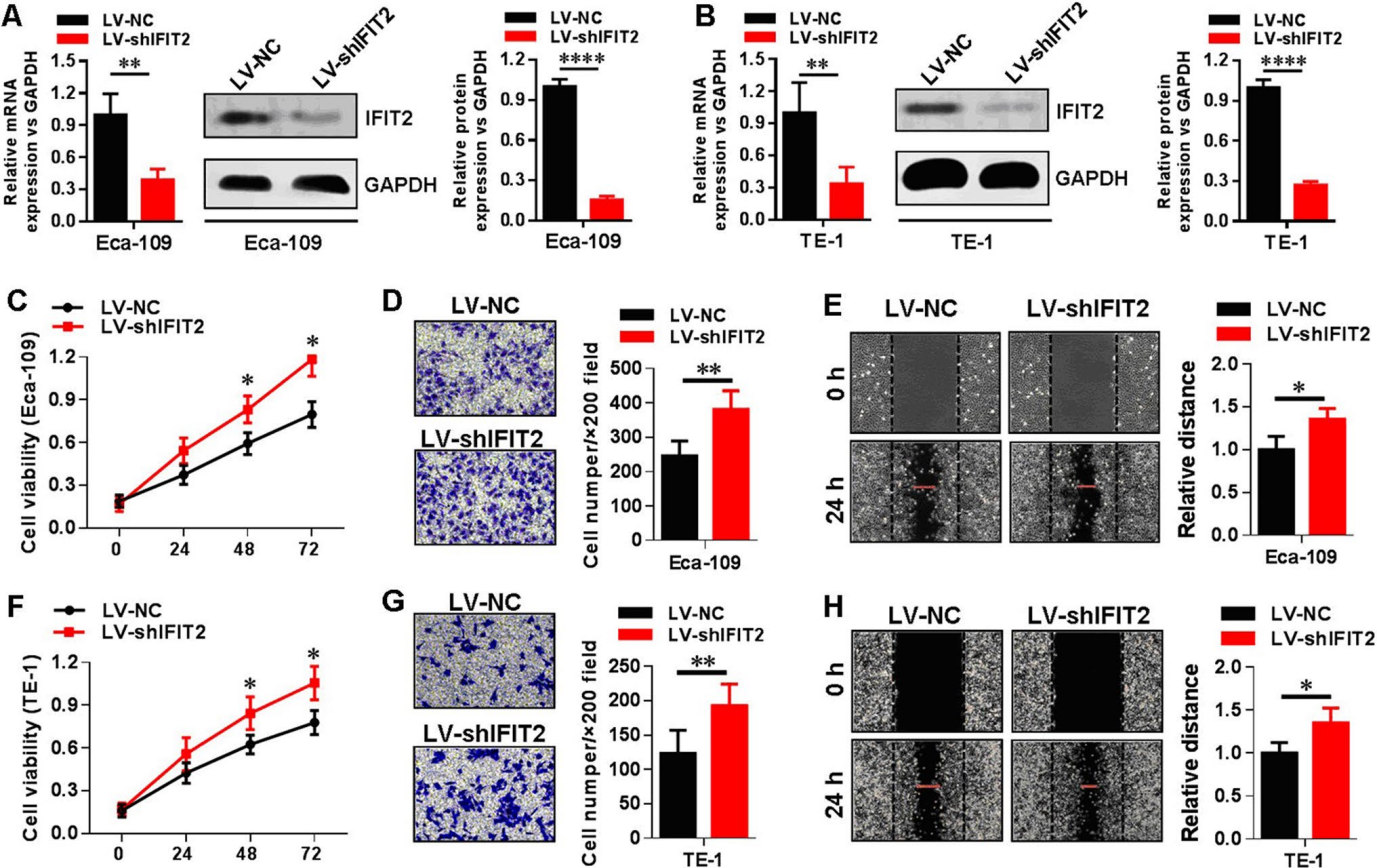
Knockdown of IFIT2 expression in human esophageal cancer cell lines. **A** and **B** In Eca-109 and TE-1 cells, after knockdown expression of IFIT2 using RNAi method, both mRNA (*P* < 0.01) and protein (*P* < 0.0001) levels of IFIT2 expression were significantly decreased compared with LV-NC control cells, respectively. **C** and **F** CCK8 results showed that at the timepoints of 48 h and 72 h, the cell viabilities of LV-shIFIT2 group cells were significantly increased compared with LV-NC group cells (both in Eca-109 and TE-1 cells, *P* < 0.05, respectively). **D** and **G** Transwell assay results showed that at the timepoint of 24 h, the cell invasion ability of LV-shIFIT2 group cells were significantly increased compared with LV-NC group cells (both in Eca-109 and TE-1 cells, *P* < 0.01, respectively). **E** and **H** Wound healing assay results showed that at the timepoint of 24 h, the cell invasion ability of LV-shIFIT2 group cells were significantly increased compared with LV-NC group cells (both in Eca-109 and TE-1 cells, *P* < 0.05, respectively)

In our present study, we established the cellular model of stable knockdown IFIT2 expression in human esophageal cancer cell lines, Eca-109 and TE-1, according to the methods we have used and described in our previous report [12, 15]. As shown in Fig. 3A, in Eca-109 cells, after knockdown expression of IFIT2 using RNAi method, at both mRNA (*P* < 0.01) and protein (*P* < 0.0001) levels, IFIT2 expression significantly decreased in contrast to the LV-NC control cells. The similar results were also found in TE-1 cells (Fig. 3B). Moreover, we further investigated the cellular functions of esophageal cancer cells after IFIT2 knockdown expression. As shown in Fig. 3C, F, the CCK8 results showed that at the timepoints of 48 h and 72 h, the cell viabilities of LV-shIFIT2 group cells were significantly increased compared with LV-NC group cells (both in Eca-109 and TE-1 cells, *P* < 0.05, respectively). As shown in Fig. 3D, G, the transwell assay results showed that at the timepoint of 24 h, the cell invasion abilities of LV-shIFIT2 group cells were significantly increased compared with LV-NC group cells (both in Eca-109 and TE-1 cells, *P* < 0.01, respectively). As shown in Fig. 3E, H, the wound healing assay results showed that at the timepoint of 24 h, the cell invasion abilities of LV-shIFIT2 group cells were significantly increased compared with LV-NC group cells (both in Eca-109 and TE-1 cells, *P* < 0.05, respectively).

### Rescue experiment in LV-shPD-L1 cells using IFIT2 knockdown expression and JAK/STAT pathway inhibition

**Fig. 4:**
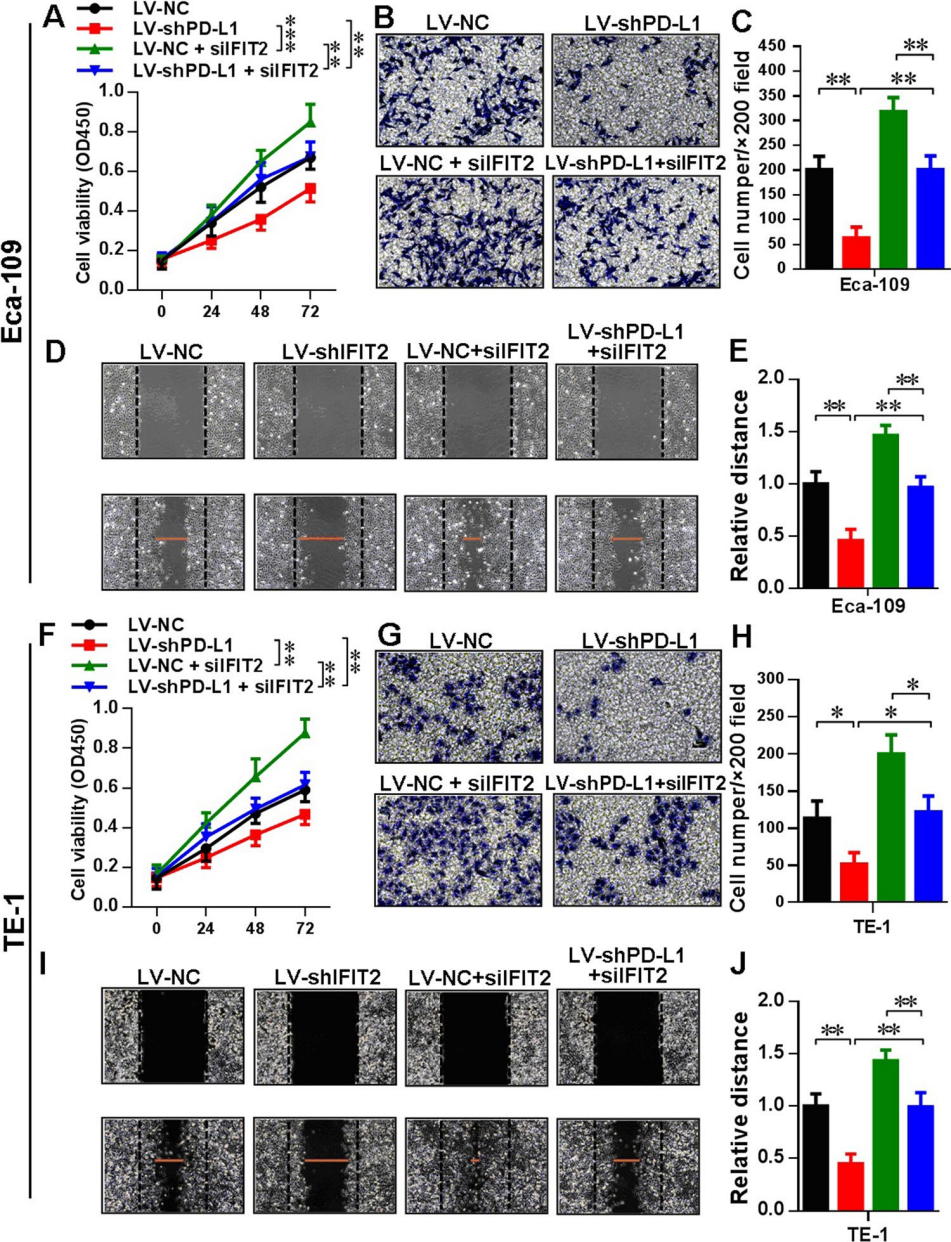
Rescue experiment in LV-shPD-L1 cells using IFIT2 knockdown expression. **A** CCK8 assay indicated that in LV-shPD-L1 cells, the cell viability significantly increased upon IFIT2 knockdown expression in Eca-109 (*P* < 0.01). **B** and **C** Transwell assay indicated that in LV-shPD-L1 cells, the cell invasion ability significantly increased upon IFIT2 knockdown expression in Eca-109 (*P* < 0.01), and the cell invasion ability of LV-shPD-L1 + siIFIT2 group also significantly lower than that in LV-NC + siIFIT2 group in Eca-109 cells (*P* < 0.01). **D** and **E** Wound healing assay indicated that in LV-shPD-L1 cells, the cell migration ability significantly increased upon IFIT2 knockdown expression in Eca-109 cells (*P* < 0.01), and the cell migration ability of LV-shPD-L1 + siIFIT2 group also significantly lower than that in LV-NC + siIFIT2 group in Eca-109 cells (*P* < 0.01). **F** CCK8 results indicated that in LV-shPD-L1 cells, the cell viability significantly increased upon IFIT2 knockdown expression in TE-1 cells (*P* < 0.01). **G** and **H** Transwell assay results indicated that in LV-shPD-L1 cells, the cell invasion ability significantly increased upon IFIT2 knockdown expression in TE-1 cells (*P* < 0.05), and the cell invasion ability of LV-shPD-L1 + siIFIT2 group also significantly lower than that in LV-NC + siIFIT2 group in TE-1 cells (*P* < 0.05). **I** and **J** Wound healing assay indicated that in LV-shPD-L1 cells, the cell migration ability significantly increased upon IFIT2 knockdown expression in TE-1 cells (*P* < 0.01), and the cell migration ability of LV-shPD-L1 + siIFIT2 group was also significantly lower than that in TE-1 cells (*P* < 0.01)

As demonstrated in Fig. 1, we found that upon PD-L1 knockdown expression, the IFIT2 expression was significantly increased. Therefore, we aimed to study the effect of IFIT2 knockdown expression on cellular functions in LV-shPD-L1 cells in contrast to LV-NC cells. As shown in Fig. 4A, F, the CCK8 results indicated that in LV-shPD-L1 cells, the cell viability significantly increased upon IFIT2 knockdown expression in Eca-109 (*P* < 0.01) and TE-1 cells (*P* < 0.01), respectively. As shown in Fig. 4B, C, G, H, the transwell assay results indicated that in LV-shPD-L1 cells, the cell invasion ability significantly increased upon IFIT2 knockdown expression in Eca-109 (*P* < 0.01) and TE-1 cells (*P* < 0.05), respectively; meanwhile, the cell invasion ability of LV-shPD-L1 + siIFIT2 group still significantly lower than that in LV-NC + siIFIT2 group (Eca-109: *P* < 0.01, and TE-1: *P* < 0.05, respectively). As shown in Fig. 4D, E, I, J, the wound healing assay results indicated that in LV-shPD-L1 cells, the cell migration ability significantly increased upon IFIT2 knockdown expression in Eca-109 (*P* < 0.01) and TE-1 cells (*P* < 0.01), respectively, and meanwhile, the cell migration ability of LV-shPD-L1 + siIFIT2 group still significantly lower than that in LV-NC + siIFIT2 group (Eca-109: *P* < 0.01, and TE-1: *P* < 0.01, respectively).

**Fig. 5:**
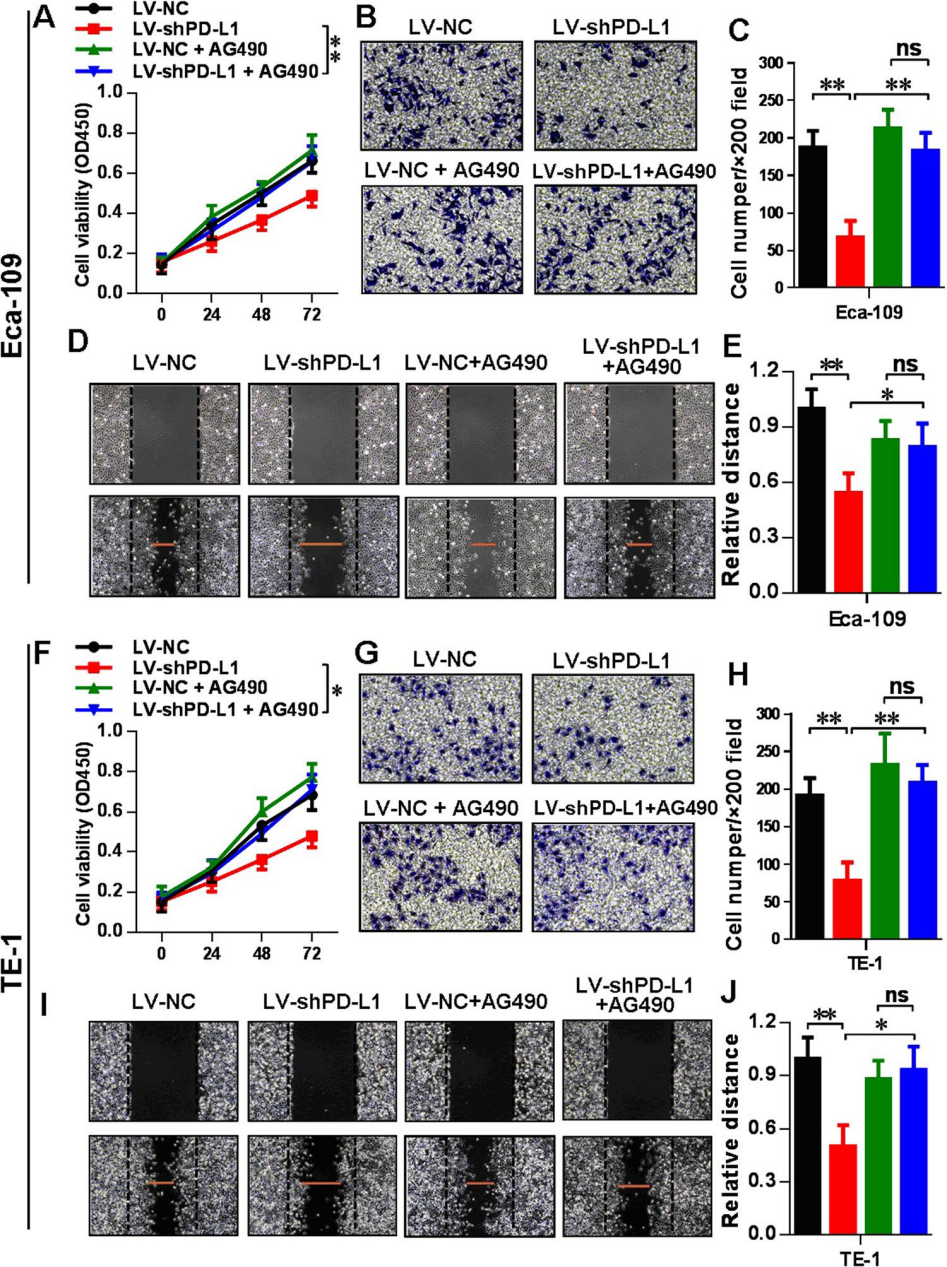
Rescue experiment in LV-shPD-L1 cells using JAK/STAT pathway inhibition. **A** CCK8 assay showed that in LV-shPD-L1 cells, the cell viability significantly increased upon AG490 treatment in Eca-109 cells (*P* < 0.01). **B** and **C** Transwell assay showed that in LV-shPD-L1 cells, the cell invasion ability was significantly increased upon AG490 treatment in Eca-109 cells (*P* < 0.01). **D** and **E** Wound healing assay indicated that in LV-shPD-L1 cells, the cell migration ability was significantly increased upon AG490 treatment own expression in Eca-109 cells (*P* < 0.05). **F** CCK8 assay showed that in LV-shPD-L1 cells, the cell viability significantly increased upon AG490 treatment in TE-1 cells (*P* < 0.01). **G** and **H** Transwell assay showed that in LV-shPD-L1 group, the cell invasion ability significantly increased upon AG490 treatment in TE-1 cells (*P* < 0.01). **I** and **J** Wound healing assay indicated that in LV-shPD-L1 group, the cell migration ability significantly increased upon AG490 treatment own expression in TE-1 cells (*P* < 0.05)

**Fig. 6:**
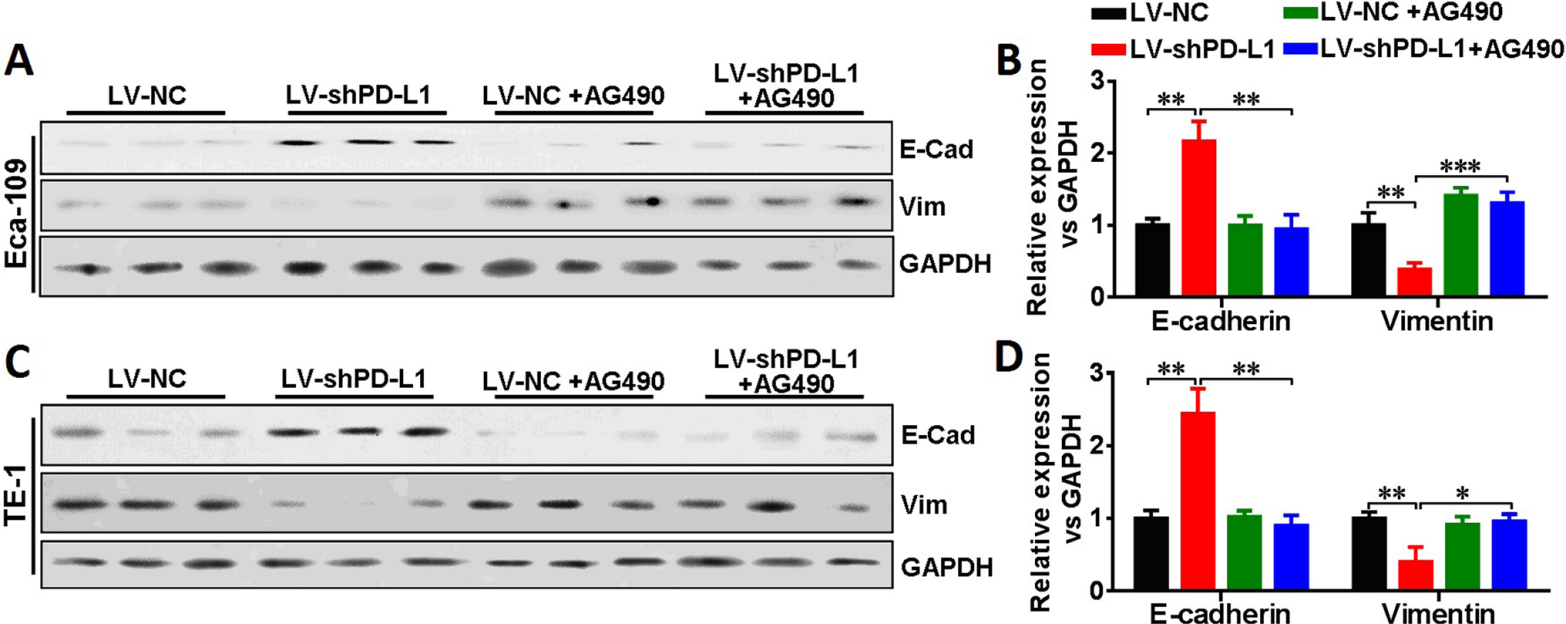
Examination of EMT markers in rescue experiment in LV-shPD-L1 cells using JAK/STAT pathway inhibition. **A** and **C** EMT markers, E-cadherin and Vimentin, were examined using Western blotting in LV-NC, LV-shPD-L1, LV-NC + AG490 and LV-shPD-L1 + AG490 groups in Eca-109 as well as TE-1 cells, respectively. **B** Western blotting results showed that the E-cadherin level was significantly decreased (*P* < 0.01), and Vimentin level was significantly increased (*P* < 0.001), upon AG490 treatment when PD-L1 was knockdown in Eca-109 cells. **D** Western blotting results showed that the E-cadherin level was significantly decreased (*P* < 0.01), and Vimentin level was significantly increased (*P* < 0.05), upon AG490 treatment when PD-L1 was knockdown in TE-1 cells

Moreover, to further confirm whether the up-stream signaling pathway of IFIT2, namely, JAK/STAT was involved in the regulation of the cellular function upon PD-L1 knockdown expression, we selected the JAK inhibitor AG490 to treat the esophageal cancer cell lines and compare the cellular functions as well as EMT phenotypes between LV-NC and LV-shPD-L1 groups. As shown in Fig. 5A, F, the CCK8 results indicated that in LV-shPD-L1 group, the cell viability significantly increased upon AG490 treatment in Eca-109 (*P* < 0.01) and TE-1 cells (*P* < 0.05), respectively. As shown in Fig. 5B, C, G, H, the transwell assay results indicated that in LV-shPD-L1 group, the cell invasion ability significantly increased upon AG490 treatment in Eca-109 (*P* < 0.01) and TE-1 cells (*P* < 0.01), respectively. As shown in Fig. 5D, E, I, J, the wound healing assay results indicated that in LV-shPD-L1 group, the cell migration ability significantly increased upon AG490 treatment own expression in Eca-109 (*P* < 0.05) and TE-1 cells (*P* < 0.05), respectively. Then, the Western blotting results showed that the E-cadherin level significantly decreased upon AG490 treatment when PD-L1 was knockdown in both Eca-109 (Fig. 6A, B , *P* < 0.01) and TE-1 cells (Fig. 6C, D , *P* < 0.01), respectively, and the Vimentin level significantly increased upon AG490 treatment when PD-L1 was knockdown in both Eca-109 (Fig. 6A, B , *P* < 0.001) and TE-1 cells (Fig. 6C, D , *P*  < 0.05), respectively. Therefore, in combination with the results from Fig. 1, first, we found that STAT1/IFIT2 signaling pathway was upregulated when PD-L1 was knockdown, and the rescue experiment in LV-shPD-L1 cells using IFIT2 knockdown expression and JAK/STAT pathway inhibition could reverse the changes of the cellular functions and EMT phenotypes.

### PD-L1 knockdown could enhance the promoter activity of *IFIT2* in esophageal cancer cells

**Fig. 7:**
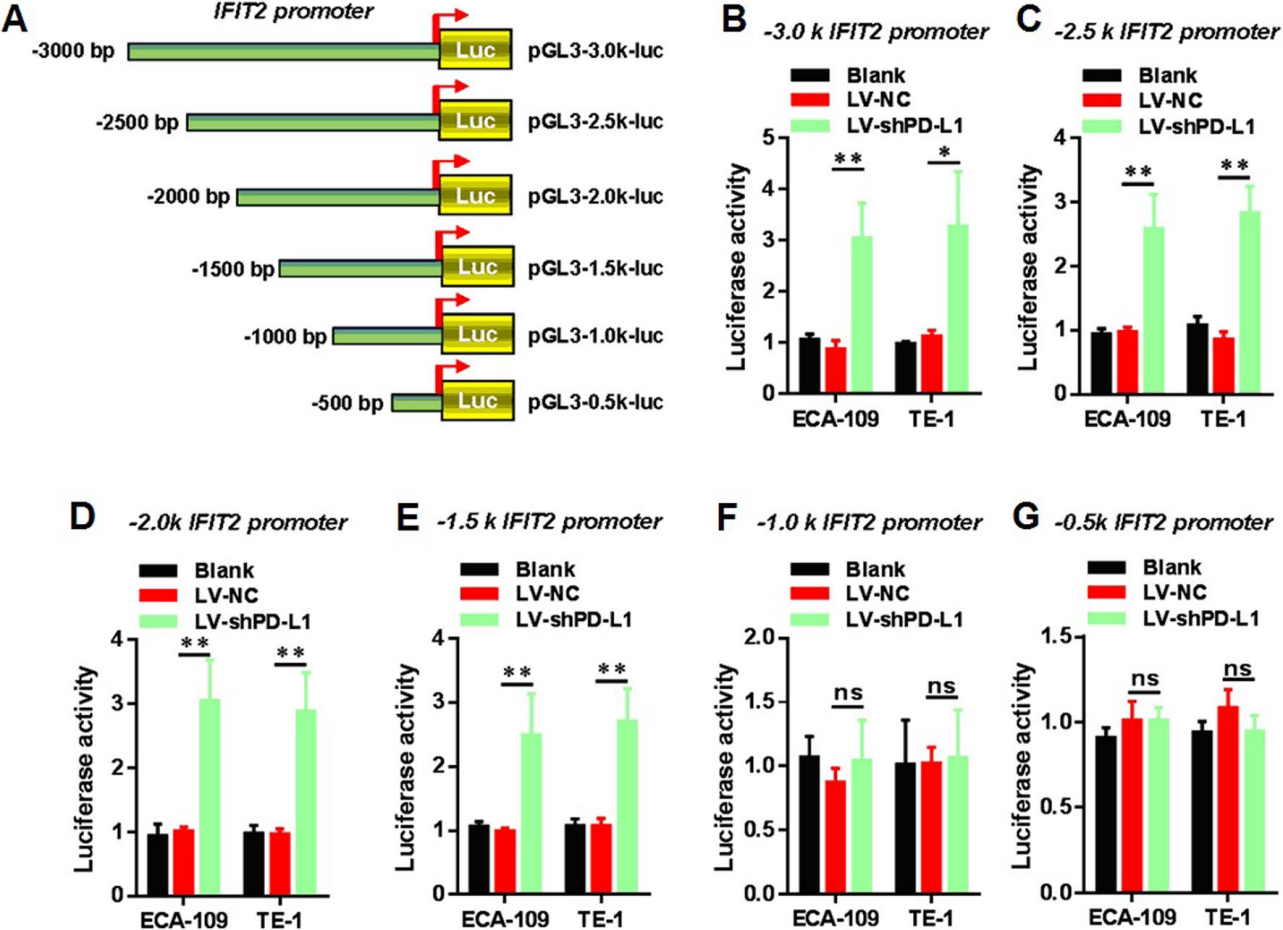
PD-L1 knockdown could enhance the promoter activity of *IFIT2* in esophageal cancer cells. **A** Six fragments of the IFIT2 promoter region were amplified and cloned into the pGL3-Basic vector. These constructed plasmids were named as pGL3-0.5 k-luc (500 bp), pGL3-1.0 k-luc (1000 bp), pGL3-1.5 k-luc (1500 bp), pGL3-2.0 k-luc (2000 bp), pGL3-2.5 k-luc (2500 bp), and pGL3-3.0 k-luc (3000 bp) according to their sequence lengths. **B**–**E** Activities of − 3000 to − 1000 region of IFIT2 promoter remained higher in LV-shPD-L1 cells compared with the LV-NC cells in both Eca-109 and TE-1 cells. **F** and **G.** Activities of – 1000–0 region of IFIT2 promoter remained no significant difference in LV-shPD-L1 cells compared with LV-NC in both Eca-109 and TE-1 cells

Based on the above-mentioned results, it was suggested that STAT1/IFIT2 signaling pathway was involved in PD-L1 mediated EMT in human esophageal cancer cells. However, the detailed mechanism was still remains elusive. Therefore, it is necessary to further explore how PD-L1 reflects the IFIT2 expression in human esophageal cancer cells needs to be studied. In the present study, we further carried out the luciferase reporter assays to examine the IFIT2 promoter activity upon knockdown expression of PD-L1 to identify the putative targeted region of IFIT2 promoter. Six fragments of the IFIT2 promoter region were amplified and cloned into the pGL3-Basic vector. These constructed plasmids were named as pGL3-0.5 k-luc (500 bp), pGL3-1.0 k-luc (1000 bp), pGL3-1.5 k-luc (1500 bp), pGL3-2.0 k-luc (2000 bp), pGL3-2.5 k-luc (2500 bp), and pGL3-3.0 k-luc (3000 bp) according to their sequence lengths (Fig. 7A). Then these plasmids were co-transfected with the pRL-TK plasmid into cells. As shown in Fig. 7B–E, we found that the activity of – 3000 to − 1000 region of IFIT2 remains higher in the LV-shPD-L1 group compared with the LV-NC group in both Eca-109 and TE-1 cells, but not within the – 1000–0 region of IFIT2 promoter (Fig. 7F, G).

## Discussion

Many previous studies have confirmed that the co-stimulatory molecule PD-L1 can induce EMT and maintain cancer cell stemness in several human cancers, thus playing an important role in promoting cancer progression [3, 6, 17–22]. Moreover, Azuma et al*.* have established the theory that PD-L1 can serve as a bidirectional regulator, the extra-cellular domain of PD-L1 can interact with its receptor PD-1 on T cells, leading to the dampening of T cell-mediated anti-tumor response, and the cytoplasmic domain of PD-L1 can trigger the signaling pathways involved in the cancerous transformation upon ligation with PD-1 fusion protein [4]. However, although some reports have shown that signaling pathways, such as PI3K/AKT, are involved in the regulation of PD-L1 mediated EMT [7], the detailed molecular mechanism of how abnormal expression of PD-L1 regulates the EMT in cancer cells still remains largely unexplored.

In our previous study, we have successfully established cellular models, including PD-L1 knockdown expression, full-length PD-L1 over-expression and cytoplasmic PD-L1 truncated over-expression in human esophageal cancer cell line Eca-109 [6]. In addition, our results have illustrated that, over-expression of PD-L1 could promote EMT of Eca-109 cells and the cytoplasmic domain of PD-L1 plays a decisive role in the driving of EMT. Based on these cellular models, we subsequently aimed to study the potential mechanism of how PD-L1 regulated EMT in esophageal cancer.

It is well known that extrinsic IFNγ can up-regulate the expression of PD-L1 in tumor cells [23]. Moreover, Fernando et al. have reported that over-expression of EGFR is significantly correlated with JAK2 and PD-L1 expression in head and neck cancer (HNC) tissues in a large cohort of HNC specimens, and PD-L1 expression was induced in an EGFR- and JAK2/STAT1-dependent manner [24]. All these results describe the scenarios that the extrinsic IFNγ stimulation and the intrinsic JAK/STAT signaling pathway are involved in the up-regulation of PD-L1 expression in cancer cells. In our present study, we focused on the intrinsic regulation of downstream signal pathway upon PD-L1 intervention. First, we detected the expression levels of STAT1 and IFIT2 in different cellular models upon PD-L1 intervention in Eca-109 cells, and our results revealed that, the total STAT1 level was increased when PD-L1 knockdown, while no difference was found between PD-L1 over-expression and control cells. Interestingly, furthermore, we found that both pSTAT1-y701 and IFIT2 levels, were significantly increased when PD-L1 knockdown and significantly decreased when PD-L1 was over-expressed, suggesting that STAT1/IFIT2 pathway was involved in the PD-L1-mediated regulation of biological function of esophageal cancer cells, and our following cellular studies also confirmed this speculation.

IFIT2, an important member of ISG family, is also known as ISG54, and it has been reported to play an important role in anti-viral and anti-cancer effects [25]. Like other ISG family members, IFIT2 can also be transcriptionally induced by triggering the JAK/STAT signaling pathway when different types of IFNs bind to the distinct cell surface receptors [26, 27]. Moreover, the patients with higher levels of ISGs, including IFIT2, seemed to be more sensitive to immune checkpoint immunotherapy compared with the patients with lower levels of ISG expression [28, 29]. IFIT2 has been recognized as an important tumor suppressor gene in our and other’s studies, and decreased IFIT2 expression significantly increased the cellular ability of invasion and migration, and lower level of IFIT2 expression in cancer tissues can predict the poor survival of the patients [12, 15, 30]. In our present work, the cellular studies results showed that in human esophageal cancer cells, decreased IFIT2 expression significantly increased the cellular ability of viability, invasion and migration. In addition, the retrospective study by examining the IFIT2 expression in human esophageal cancer tissues also demonstrated that decreased IFIT2 expression could be used as an independent prognostic predictor for esophageal cancer patients.

Because of STAT1 as well as IFIT2 was up-regulated in PD-L1 knockdown expression cells in contrast to control cells, we also performed the rescue experiment and further identified that either STAT1 inhibition or IFIT2 knockdown expression in LV-shPD-L1 cells could reverse the phenotypes caused by PD-L1 knockdown expression. Moreover, we also performed the luciferase reporter assay, and further confirmed that in both Eca-109 and TE-1 cells, the promoter region of IFIT2 (− 3 to − 1 K) remained more active in LV-shPD-L1 cells in contrast to LV-NC cells, suggesting that over-expression of PD-L1 could inhibit the promoter activity of IFIT2 and then down-regulate the IFIT2 expression, leading to the cancerous transformation and EMT. Collectively, our current findings provided a novel mechanism underlying the effects of PD-L1 on EMT of cancers, showing that STAT1/IFIT2 signaling pathway was required in PD-L1-mediated EMT in human esophageal cancer.

## Conclusion

Our present findings provided a novel mechanism underlying the effects of PD-L1 on EMT of cancer cells, showing that STAT1/IFIT2 signaling pathway was required in PD-L1-mediated EMT in human esophageal cancer.

## Data Availability

The data sets supporting the conclusions of this article are included within the article.

## Abbreviations

PD-L1: Programmed cell death-Ligand 1
ISGs: Interferon-stimulated genes
EMT: Epithelial-to-mesenchymal transition
OS: Overall survival
IHC: Immunohistochemistry
RT-PCR: Real-time polymerase chain reaction
CCK-8: Cell Counting Kit-8
